# Internet Queries and Methicillin-Resistant *Staphylococcus aureus* Surveillance

**DOI:** 10.3201/eid1706.101451

**Published:** 2011-06

**Authors:** Vanja M. Dukic, Michael Z. David, Diane S. Lauderdale

**Affiliations:** Author affiliations: University of Colorado, Boulder, Colorado, USA (V.M. Dukic);; University of Chicago, Chicago, Illinois, USA (M.Z. David, D.S. Lauderdale)

**Keywords:** methicillin-resistant Staphylococcus aureus, MRSA, bacteria, antimicrobial resistance, Internet protocol, surveillance, Google Trends, Internet, dispatch

## Abstract

The Internet is a common source of medical information and has created novel surveillance opportunities. We assessed the potential for Internet-based surveillance of methicillin-resistant *Staphylococcus aureus* and examined the extent to which it reflects trends in hospitalizations and news coverage. Google queries were a useful predictor of hospitalizations for methicillin-resistant *S. aureus* infections.

*Staphylococcus aureus* is the most common bacterial pathogen isolated from human infections (*1*). Methicillin-resistant *Staphylococcus aureus* (MRSA) isolates are strains constitutively resistant to β-lactam antimicrobial drugs. MRSA was initially largely confined to patients with health care exposures (*2*), but in the late 1990s, genetically distinct strains emerged and spread rapidly among healthy persons in the United States. These new strains, known as community-associated MRSA (CA-MRSA), differ epidemiologically and genetically from older strains (*2**,**3*). CA-MRSA strains have become the most common cause of skin infections in US emergency departments (*4*).

There is no systematic surveillance system in the United States for MRSA. The Centers for Disease Control and Prevention (CDC) tracks a limited group of infections defined as invasive through the Active Bacterial Core (ABC) surveillance system reported from 9 regions. These include MRSA infections at normally sterile sites. In a 2007 report, CDC used ABC surveillance to estimate that there were 94,000 cases and 18,650 deaths caused by invasive MRSA disease in the United States in 2005 (*5*). This report received extensive media coverage and increased public awareness of MRSA (*6*).

Recent efforts to overcome surveillance limitations, in particular delay and limited geographic coverage, have included Internet protocol (IP) surveillance. IP surveillance monitors Internet search terms related to a specific disease, assuming that greater disease activity correlates with more searches. The best known IP surveillance is Google Flu Trends (*7*), although other researchers have created additional models (*8**,**9*). Given the lack of comprehensive surveillance, we examined whether Google search data might productively supplement existing systems to track the changing epidemiology of MRSA infections. Because MRSA, unlike influenza, is unfamiliar to many persons, we hypothesized that Internet search activity might reflect curiosity inspired by news reports and information-seeking related to actual infections or symptoms.

## The Study

We used the Google Trends database to obtain the proportion of all Google searches that contained the words “MRSA” or “staph.” “Staph” was included because many news stories refer to MRSA as “antibiotic resistant staph.” “Methicillin-resistant *Staphylococcus aureus*” was too infrequently searched to be useful. Google Trends reports search activity relative to the average number of similar queries in February 2004. We only included US searches determined from IP addresses.

We extracted counts of US newspaper, wire service, and radio and television stories mentioning “MRSA” or “staph” from the LexisNexis Academic database. We spot-checked stories with the word “staph” to confirm they were about MRSA. One event or medical publication could generate multiple news stories. We hypothesized that the volume of news coverage captured the relative effect of the story on search behavior.

We used quarterly hospital discharge data from the University HealthSystems Consortium Clinical Database, which includes >90% of US academic medical centers, to calculate the proportion of hospitalizations including an MRSA diagnosis. These data were a proxy for true MRSA incidence. We used the diagnostic code for MRSA from the International Classification of Disease, 9th Revision (V09.0). MRSA hospitalizations include CA-MRSA infections that led to hospitalization and infections that developed during a hospitalization. This database includes <99 codes per discharge, more than other national hospital discharge databases. The likelihood of recording an MRSA diagnosis increases with longer lists of codes because of the many concurrent conditions in complex hospitalizations. Some medical centers systematically used <99 diagnoses fields. We adjusted hospitalization rates for the maximum number of codes submitted by each medical center each year. Data after the 3rd quarter of 2008 were not included because of implementation of a nationwide coding change for MRSA.

We related quarterly variation in MRSA hospitalizations to quarterly variations in search queries and news stories in a linear regression model. Because of the effect of the 2007 CDC report on MRSA awareness, we tested 2 indicator variables: 1 to capture the spike in search activity during the 4th quarter of 2007, and 1 to account for higher levels of search activity in subsequent quarters (*10*). These 2 indicators enable the model baseline to differ during the quarters before, during, and after the 4th quarter of 2007, while keeping the relationship between hospitalization rates and Internet searches and news counts the same during the 3 periods. All statistical analyses were performed in Stata version 10.0 (StataCorp LP, College Station, TX, USA).

**Figure 1:**
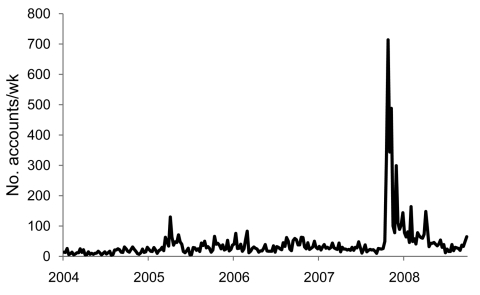
Weekly counts of news coverage (newspaper stories, wire service stories, and television and radio news transcripts) that mention “MRSA” (methicillin-resistant *Staphylococcus aureus*) or “staph,” 2004–2008. Extracted from the LexisNexis Academic Database.

Details of the model and statistical methods are available in the Technical Appendix. Weekly news counts are shown in Figure 1. They range from 4 to 130 before the October 2007 peak of 719, related to the CDC report, the effect of which appears to linger. The prior peak of 130 in April 2005 was related to articles in the New England Journal of Medicine describing necrotizing fasciitis associated with MRSA and the emergence of CA-MRSA in 2001–2002 (*11**,**12*).

**Figure 2:**
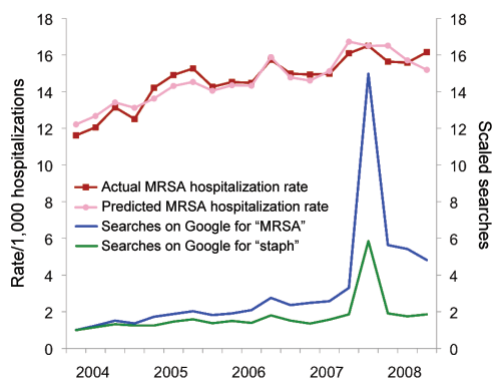
Actual and predicted hospitalization rates per 1,000 hospitalizations with an International Classification of Disease, 10th Revision, diagnostic code for methicillin-resistant *Staphylococcus aureus* (MRSA) and the fraction of Google search queries for “MRSA” or “Staph” (relative to the fraction of February 2004), 2004–2008.

Quarterly variation in Google searches for “MRSA” and “staph” are shown in Figure 2. Search behavior changed markedly after the October 2007 publication. In addition to the spike, there was a subsequent change in the relative frequency of search term “MRSA” compared with “staph.” Note that the news count peak in 2005 is not seen in the Google searches, and the peak in the Google searches in the 3rd quarter of 2006 is not apparent in the news counts.

Google queries were a useful predictor of MRSA hospitalizations and explained 33% of quarterly variation when used alone. Adding news counts to the model resulted in increasing the percentage of explained variation only modestly to 41%. The news counts were not a significant addition to the model (p = 0.18).

Our final model, which includes search queries and the 2 temporal indicator variables, but not the news counts, is shown in the Table. The correlation between model predictions and observed hospitalization rates was 0.93 (p<0.001). Although data after 2007 are insufficient for definitive comparison, a better prediction before than after the 4th quarter of 2007 is suggested (Figure 2).

**Table:** Multiple regression results for model relating UHC MRSA hospitalization rates per 1,000 hospitalizations to Google searches for “MRSA” or “staph” (normalized and scaled)*

Characteristic	Coefficient	95% CI	SE	t value	p>t
Intercept	9.03	7.56 to 10.50	0.69	13.07	<0.001
Google searches	0.25	0.18 to 0.32	0.032	7.73	<0.001
2007 4th quarter indicator	−21.45	−28.10 to −14.80	3.12	−6.87	0.001
2008 indicator	−3.06	−4.55 to −1.57	0.70	−4.37	<0.001

*UHC, University HealthSystems Consortium; MRSA, methicillin-resistant *Staphylococcus aureu*s; CI, confidence interval. The overall model F(3,15) was 29.69 (p<0.0001), R^2^ 0.8559, and adjusted R^2^ 0.8270. Correlation coefficient between predicted values of this model and observed rates was 0.9251.

## Conclusions

We report an IP surveillance model for MRSA incidence. We hypothesized that news coverage for such an unfamiliar disease would strongly influence search activity. However, news coverage did not affect the relationship between search queries and hospitalization rates before the 2007 CDC report. The congruence of the Internet search activity and the hospital discharge data suggest that their temporal pattern represents the actual trend in MRSA: an increasing incidence during 2004–2007, with a suggestion of seasonal variation, and no increase in 2008. This pattern is not the same pattern documented by the ABC surveillance data for invasive MRSA infections (*13*).

The unfamiliarity of the public with MRSA poses a challenge to using Google Trends. Searches using the phonetic misspelling “mersa” show a parallel trend to searches using “MRSA,” although they are less frequent, and the correctly spelled “methicillin” is too rare to track.

Hospitalized MRSA infections include hospital-associated MRSA infections and the more serious CA-MRSA infections. Because evidence has shown that invasive hospital-associated MRSA infections decreased during the study period (*13*), the generally upward secular trend in MRSA hospitalizations is more likely to represent the trend in CA-MRSA, especially because we now know that most MRSA infections have onset in the community (*3*). The inability to distinguish community and health care infections is nonetheless a limitation of the Google and the hospitalization data. Although some hospital databases include more hospitals, they include fewer diagnostic codes. Therefore, there are no additional comprehensive data available for MRSA incidence. The lack of any true standard for MRSA incidence is why IP surveillance is potentially useful.

## Supplementary Material

Technical Appendix Analysis information and an appendix table on multiple regression results for the model.
