# Supplementary material for: Internet Queries and Methicillin-Resistant Staphylococcus aureus Surveillance
Source: Emerg Infect Dis. 2011 Jun;17(6):1068–70. doi: 10.3201/eid1706.101451 (PMC3358097; doi:10.3201/eid1706.101451)
Supplement: Technical Appendix — Analysis information and an appendix table on multiple regression results for the model. [file 10-1451-Techapp.pdf]

Article DOI: 10.3201/eid1706.101451

Internet Queries and Methicillin-Resistant *Staphylococcus aureus* Surveillance

Technical Appendix

Analysis

Relating temporal variation in methicillin-resistant *Staphylococcus aureus* (MRSA) diagnoses to temporal variation in search queries over time (*t*) was first conducted by using a linear regression model

$$H_t = b_0 + b_1 Q_t + b_2 N_t + e_t \quad (1)$$

where H_t denotes the quarterly proportion of hospitalization discharges from 2004 to 2008 that contained a diagnostic code for MRSA, and Q_t denotes the fraction of Google search queries that contained the words "MRSA" and/or "Staph." Because MRSA, in contrast to influenza, is unfamiliar to many persons, we hypothesized that Internet search activity might reflect curiosity inspired by news reports and information-seeking related to actual individual infections or symptoms. To test this hypothesis, we include a second predictor, N_t, the quarterly counts of news stories extracted from the LexisNexis database.

In the above model, the coefficient b_0 denotes the average MRSA hospitalization discharge rate in the absence of any MRSA-related Internet search and news activity. Conversely, b_1 and b_2 denote the adjusted effects of the search activity and news stories, respectively, on the average quarterly MRSA hospitalization discharge rates. The error term, e_t, denotes the residual random variation in the hospitalization rates that is uncorrelated with the quarterly Internet search activity or news counts. The errors might be correlated over time, and we discuss this issue specifically toward the end of this Appendix.

Because of the effect of the 2007 Centers for Disease Control and Prevention report on awareness of MRSA among the general public, we also consider adding 2 indicator variables to the model: one to eliminate the large spike in search activity during the 4th quarter of 2007

because of this report, and one to account for the possibly increased baseline search activity level as a result of this report in the subsequent quarters. The expanded linear regression model is then

$$H_t = c_0 + c_1 Q_t + c_2 N_t + c_3 I_t + c_4 P_t + e_t \quad (2).$$

The indicator I_t is on (set to 1) only in the last quarter of 2007, and P_t is set to 1 only in post-2007 quarters. These 2 indicators enable the model baseline to differ during the quarters before, during, and after the 4th quarter of 2007. However, this model keeps the relationship between MRSA Internet searches and hospitalization rates, and between news counts and hospitalization rates, the same during the 3 periods. Thus, the coefficient c_1 describes the basic relationship between Internet activity and MRSA incidence, adjusted for the media effect stemming from news stories about MRSA and the 2007 Centers for Disease Control and Prevention report.

Finally, as with any data recorded over time, autocorrelation of the errors over time might be a potential problem. To test for the presence of autocorrelation, a Prais-Winsten transformed regression was conducted simultaneously, but little difference between the original model and the transformed model was observed. The 2 models were qualitatively similar and had nearly identical predicted values. Because our purpose focused on prediction of MRSA incidence, and not inference, we chose to present the simpler model.

Supplementary Results

The model presented in the Table in the main text of the paper shows that the effects of the 2 indicators are estimated to be negative. Had we omitted the 2 indicators, the predicted hospitalization rate in those quarters would have been high because of the high level of search activity. Thus, negative coefficients during the spike quarters eliminate the overestimate of MRSA incidence in those periods.

For completeness, we also present the Table in this technical appendix, which shows results from the model that also include news counts in addition to the predictors from the Table in the main text. This model resulted in similar statistics (overall $F[4,14]$ 20.87, $p<0.001$, adjusted R^2 0.815), increasing the correlation between the model predictions and the observed hospitalization rates negligibly from 0.9251 to 0.9254.

Technical Appendix Table. Multiple regression results for model relating UHC MRSA hospitalization rates per 1,000 persons to Google searches for “MRSA” or “staph (normalized and scaled)*

Characteristic	Coefficient	Standard error	*t* value	p>*t*	95% CI
Intercept	9.04	0.71	12.65	<0.001	(7.50 to 10.57)
Google searches	0.25	0.04	6.24	<0.001	(0.16 to 0.33)
News counts	0.0003	0.0012	0.23	0.825	(–0.002 to 0.003)
2007 4th quarter indicator	–21.66	3.37	–6.43	0.001	(–28.88 to –14.44)
2008 indicator	–3.05	0.72	–4.22	<0.001	(–4.60 to –1.50)

*UHC, University HealthSystems Consortium; MRSA, methicillin-resistant *Staphylococcus aureus*; CI, confidence interval. The overall model F(4,14) was 20.87 (p<0.0001), R^2 0.8564, and adjusted R^2 0.8154. Correlation coefficient between predicted values of this model and the observed rates was 0.9254.
